# Risk prediction and interaction analysis using polygenic risk score of type 2 diabetes in a Korean population

**DOI:** 10.1038/s41598-024-55945-2

**Published:** 2024-03-21

**Authors:** Minsun Song, Soo Heon Kwak, Jihyun Kim

**Affiliations:** 1 Department of Statistics & Research Institute of Natural Sciences, Sookmyung Women’s University, Seoul, 04310 Korea; 2 Department of Internal Medicine, Seoul National University Hospital, Seoul, 03080 Korea; 3 Department of Statistics, Sookmyung Women’s University, Seoul, 04310 Korea

**Keywords:** Endocrinology, Statistics

## Abstract

Joint modelling of genetic and environmental risk factors can provide important information to predict the risk of type 2 diabetes (T2D). Therefore, to predict the genetic risk of T2D, we constructed a polygenic risk score (PRS) using genotype data of one Korean cohort, KARE (745 cases and 2549 controls), and the genome-wide association study summary statistics of Biobank Japan. We evaluated the performance of PRS in an independent Korean cohort, HEXA (5684 cases and 35,703 controls). Individuals with T2D had a significantly higher mean PRS than controls (0.492 vs. − 0.078, *p* $\approx 0$). PRS predicted the risk of T2D with an AUC of 0.658 (95% CI 0.651–0.666). We also evaluated interaction between PRS and waist circumference (WC) in the HEXA cohort. PRS exhibited a significant sub-multiplicative interaction with WC (OR_interaction_ 0.991, 95% CI 0.987–0.995, *p*_interaction_ = 4.93 × 10^–6^) in T2D. The effect of WC on T2D decreased as PRS increased. The sex-specific analyses produced similar interaction results, revealing a decreased WC effect on T2D as the PRS increased. In conclusion, the risk of WC for T2D may differ depending on PRS and those with a high PRS might develop T2D with a lower WC threshold. Our findings are expected to improve risk prediction for T2D and facilitate the identification of individuals at an increased risk of T2D.

## Introduction

Type 2 diabetes (T2D) is a complex metabolic disorder associated with an increased risk of chronic vascular complications, including coronary artery disease, stroke, retinopathy, and chronic kidney disease. It is a foremost global public health concerns^1^. Its prevalence has continuously increased over the past few decades, particularly, in underdeveloped contries^2^.

T2D is characterized by a strong genetic predisposition with estimates of heritability at 25–72%^3^. Genome-wide association studies (GWAS) have successfully identified more than 700 common genetic variants associated with the risk of T2D^4–9^. However, most of these common genetic variants discovered by GWAS have small effect sizes, and individually, have limited liability variance for T2D^10^. Thus, the polygenic risk score (PRS), which aggregates many genetic variants, has been used to quantify the genetic risk in an individual^11^.

Waist circumference (WC) reflects abdominal obesity and is a significant predictor of T2D^12^. It is a slightly better predictor of T2D than body mass index (BMI), which reflects overall adiposity^13^. Although WC and PRS are good predictors of T2D, the joint etiology of PRS and WC in T2D requires further elucidation. The PRS-WC interaction can provide important information for the joint modelling of genetic and environmental risk factors to predict T2D. Therefore, we aimed to construct a PRS for T2D using the Korean Association Resource (KARE) cohort and GWAS summary statistics of Biobank Japan^7^. We then evaluated the PRS for risk prediction of T2D and investigated whether the effect of PRS would depend on WC in an independent cohort, Health Examinees (HEXA) cohort.

## Results

The PRS constructed using KARE and Biobank Japan were evaluated in HEXA as follows.

### T2D risk prediction using PRS

The area under the receiver operating characteristic curve (AUC) of the PRS for predicting T2D was 0.658 (95% confidence interval [CI]: 0.651–0.666) (Supplementary Fig. S1a). The mean and standard deviation (SD) of the PRS in cases and controls were 0.492 (0.981) and − 0.078 (0.980), respectively (*p* $\approx$ 0) (Supplementary Fig. S2a). Moreover, PRS was significantly associated with T2D (odds ratio [OR] 1.964, 95% CI 1.901–2.028, *p* $\approx$ 0) (Table 1). The prevalence of T2D in each quartile of PRS was 6.562%, 10.642%, 14.381%, and 23.350% from the lowest to the highest quartiles, respectively. T2D cases were distributed 11.946%, 19.370%, 26.179%, and 42.505% from the lowest to highest quartiles of PRS, respectively. Compared to the reference group of first quartile of PRS, which reflected low genetic susceptibility, each PRS quartile exhibited substantial increase in the risk of T2D (OR 1.744 [95% CI 1.572–1.935, *p* = 9.06 × 10^–26^], OR 2.608 [95% CI 2.361–2.882 *p* = 2.30 × 10^–79^], OR 5.132 [95% CI 4.667–5.644, *p* = 3.86 × 10^–249^], respectively) (Table 1). A monotonic relationship was observed between the PRS quartiles and risk of T2D (*p*_trend_ = 4.48 × 10^–291^). The high-risk group (top 5%–25% PRS) had a significantly elevated risk of T2D compared to the remaining population: the top 5% PRS had a 4.192-fold risk, and the top 10% PRS had a 3.596-fold risk (Table 2). Similar results regarding the performance of PRS in the KARE cohort are shown in Supplementary Tables S1 and S2 and Supplementary Figs. S1b and S2b.

**Table 1:** Association between polygenic risk score and risk of T2D.

PRS	Controls	Cases	OR^a^	95% CI	P-value
PRS-ordinal					
Q1 (low risk)	9668	679	Ref		
Q2	9245	1101	1.744	(1.572–1.935)	9.06 × 10^–26^
Q3	8859	1488	2.608	(2.361–2.882)	2.30 × 10^–79^
Q4 (high risk)	7931	2416	5.132	(4.667–5.644)	3.86 × 10^–249^
P-trend = 4.48 × 10^–291^					
PRS-continuous	35,703	5684	1.964	(1.901–2.028)	0

^a^OR from logistic regression models were adjusted for age, sex, and BMI. *Q* quartile, *OR* odds ratio, *CI* confidence interval, *PRS* polygenic risk score, *BMI* body mass index, *T2D* type 2 diabetes.

**Table 2:** Risk in high polygenic risk score groups for T2D development.

High PRS group	Reference group	OR^a^	95% CI	P-value
Top 25%	Remaining 75%	2.937	(2.758–3.126)	4.50 × 10^–249^
Top 20%	Remaining 80%	3.041	(2.848–3.248)	3.44 × 10^–242^
Top 15%	Remaining 85%	3.250	(3.028–3.487)	5.89 × 10^–235^
Top 10%	Remaining 90%	3.596	(3.318–3.896)	4.94 × 10^–214^
Top 5%	Remaining 95%	4.192	(3.776–4.652)	9.98 × 10^–160^

^a^OR from logistic regression models were adjusted for age, sex, and BMI. *OR* odds ratio, *CI* confidence interval, *PRS* polygenic risk score, *BMI* body mass index, *T2D* type 2 diabetes.

### PRS-WC interaction

The results of the analyses of the main-effect-only model and the joint model are shown in Table 3. A larger WC was associated with an increased risk of T2D. We found a sub-multiplicative interaction between PRS and WC with respect to the risk of T2D (OR_interaction_ 0.991, 95% CI 0.987–0.995, *p*_interaction_ = 4.93 × 10^–6^). WC ≥ 90 cm in men and WC ≥ 85 cm in women is defined as abdominal obesity^14^. The risk of T2D associated with PRS differed when stratified by WC, and weaker associations were observed among individuals with abdominal obesity (Supplementary Table S3). The effect size of the association between PRS and the risk of T2D in individuals those with and without abdominal obesity was OR 1.758 (95% CI 1.665–1.855, *p* $\approx$ 0) and OR 2.083 (95% CI 2.001–2.168, *p* $\approx$ 0), respectively. We found similar results, showing a significant sub-multiplicative interaction between PRS and WC in T2D development from the analyses for the KARE cohort (Supplementary Tables S3 and S4). However, no significant additive interaction was observed between abdominal obesity and dichotomized PRS, where the low and high genetic risk groups included individuals with a PRS less than the median PRS and with a PRS larger than or equal to the median PRS, respectively. In the corresponding analysis, there was a significant sub-multiplicative interaction between abdominal obesity and dichotomized PRS. The estimated relative excess risk due to interaction (RERI) was 0.023 (95% CI − 0.298 to 0.349), while OR_interaction_ was 0.771 (95% CI 0.677–0.879).

**Table 3:** Results under the main-effect-only models and under the joint effect model incorporating interaction between PRS and WC for combined and sex-stratified analyses.

Model	OR	95% CI	P-value
All (n = 41,387)			
Main-effect-only model			
WC	1.044	(1.038–1.051)	1.42 × 10^–42^
PRS	1.964	(1.901–2.028)	0
Joint effect model incorporating interaction			
Interaction between WC and PRS	0.991	(0.987–0.995)	4.93 × 10^–6^
Men (n = 13,553)			
Main-effect-only model			
WC	1.048	(1.038–1.058)	2.19 × 10^–21^
PRS	1.939	(1.848–2.035)	2.81 × 10^–160^
Joint effect model incorporating interaction			
Interaction between WC and PRS	0.985	(0.979–0.991)	4.41 × 10^–6^
Women (n = 27,834)			
Main-effect-only model			
WC	1.041	(1.032–1.049)	9.14 × 10^–22^
PRS	1.980	(1.895–2.068)	1.62 × 10^–206^
Joint effect model incorporating interaction			
Interaction between WC and PRS	0.993	(0.987–0.998)	0.007

The main-effect-only model for WC included covariates and WC. The main-effect-only model for PRS included covariates and PRS. The joint effect model incorporating interaction included covariates, WC, PRS, and the interaction term between PRS and WC. For the combined analysis, covariates were age, BMI, and sex. For sex-stratified analyses, the covariates were age and BMI. The WC was measured three times, and the average value was used. *OR* odds ratio, *CI* confidence interval, *PRS* polygenic risk score, *WC* waist circumference, *BMI* body mass index.

### Discrimination results

We examined the discrimination ability of the PRS stratified by abdominal obesity and observed that individuals with abdominal obesity were discriminated less by the PRS than individuals without abdominal obesity. The AUC of individuals with and without abdominal obesity was 0.630 (95% CI 0.616–0.643) and 0.679 (95% CI 0.670–0.688), respectively. The results for the KARE cohort were similar to those for HEXA cohort (Supplementary Table S5).

We performed the prediction model using WC only and the WC was found to have an AUC of 0.694. Incorporating PRS with WC improved the AUC to 0.750. We evaluated the model incorporating age, sex, BMI, and WC, which showed an AUC of 0.747. The model with PRS added to age, sex, BMI, and WC had higher AUC (AUC = 0.794), than the model with age, sex, BMI, and WC. The results are provided in Supplementary Table S6.

### Sex-specific analysis

We performed interaction analyses and discrimination evaluations stratified by sex (Table 3 and Supplementary Table S5). Similar to the results of combined analysis, for men, WC was associated with an increased risk of T2D, PRS was associated with an increased risk of T2D, significant sub-multiplicative interaction between PRS and WC existed, and the discriminatory performance of PRS for individuals without abdominal obesity was better than that in individuals with abdominal obesity. For women, the results were similar to those for men.

## Discussion

In this study, we constructed a PRS for T2D based on 1004 single nucleotide polymorphisms (SNPs) in 3294 subjects in the KARE cohort using GWAS summary statistics of Biobank Japan^7^ and evaluated the PRS in 41,387 subjects from the HEXA cohort. We found that one SD increase in PRS was significantly associated with a 1.964-fold increased risk of T2D. The diagnostic accuracy of the PRS based on the AUC was 0.658. When PRS was divided into quartiles, individuals in the highest-risk group had a 5.132-fold increased risk compared to those in the lowest risk group. There was a significant multiplicative interaction between PRS and WC and PRS had a greater effect in individuals without abdominal obesity than in those with abdominal obesity. Overall, our study shows the potential utility of the PRS to stratify high-risk individuals with T2D for requiring preventive measures in the Korean population.

As our study showed, PRS has a great potential to identify and stratify individuals with risk of diseases, predict the risk of disease, and contribute to precision medicine. Because of the importance of PRS, many methods for computing PRS have been developed. The methods include clumping-and-thresholding (CT) (PRSice2^15^), Bayesian approaches (LDpred^16^ or LDpred2^17^, PRS-CS^18^, SBayesR^19^), and a penalized regression method (Lassosum^20^). CT relies on clumping and p-value thresholding to select SNPs for PRS construction. To infer the posterior mean effects of SNPs in Bayesian methods, LDpred^16^ and LDpred2^17^, assign a point-normal prior to SNP effect sizes, PRS-CS^18^ uses a continuous shrinkage prior on SNP effect sizes, and SBayesR^19^ utilizes a prior that consists of a point mass at zero along with a mixture of normal distributions. Lassosum^20^ uses lasso to select SNPs and construct PRS from GWAS summary statistics. Our PRS model constructed by one of the most commonly used PRS methods, PRSice2^15^, showed a significant association with T2D.

We showed that PRS was a strong risk factor for T2D, with an OR of 1.964. However, the diagnostic accuracy of PRS was only moderate, with an AUC of 0.658. For assessing diagnostic accuracy, an AUC above 0.70 is considered acceptable. It should be taken into account that no other clinical or environmental risk factors were included in the risk model. The PRS-only model would be similar to predicting someone’s risk of T2D only with PRS without any clinical information. It is fair to speculate that the AUC would have increased when other clinical risk factors, including age, sex, BMI, and WC, were included in the model.

There are arguments that adding PRS to clinical risk factors does not improve AUC. This is based on the fact that clinical risk model, including fasting glucose and hemoglobin A1c (HbA1c), already achieves AUC that reaches 0.90^21^. The PRS is considered to aid risk stratification and, therefore, identify high-risk individuals^22^. Our results showed that the OR for T2D in the highest PRS quartile was 5.132-fold higher than that in the lowest PRS quartile. Similarly, individuals with PRS in top 5% had 4.192-fold increased risk compared to those with PRS in the remaining 95%. As much as 34% individuals with the PRS in the top 5% had T2D. These individuals should be targeted for preventive measures and earlier screening.

We investigated the multiplicative and additive interactions between PRS and WC, a non-genetic risk factor for T2D. WC reflects abdominal obesity and is a well-known risk factor for T2D. As expected, WC was a significant predictor of T2D in both the cohorts. Although we did not find evidence of a significant additive interaction between abdominal obesity and PRS status, we observed a significant negative multiplicative interaction between PRS and WC. The genetic effect estimate of PRS was larger in individuals without abdominal obesity (smaller WC) than those with abdominal obesity (larger WC). Many of the T2D genetic risk loci are associated with decreased beta-cell function^23^. It is speculated that individuals with abdominal obesity have a higher environmental risk of T2D and the relative effect of PRS would be modest. However, for those without abdominal obesity and lower environmental risk of T2D, genetic risk as reflected in PRS would exert larger effect. This finding also suggests that non-obese individuals with T2D may have a higher genetic risk of T2D. In addition, when stratified by abdominal obesity, the discriminatory performance of PRS in terms of AUC increased for individuals without abdominal obesity compared to that for individuals with abdominal obesity. That finding implies that PRS is more important risk predictor of T2D in individuals without abdominal obesity.

The strengths of our study include the following. First, we used GWAS summary statistics derived from Japanese whose ancestry are relatively close. Second, we used two large independent cohorts to train and validate the PRS model. Lastly, we investigated the interaction between PRS and WC, a key environmental factor of T2D. However, this study has certain limitations. The main analysis in this study was based on case–control logistic regression as there was insufficient longitudinal follow-up information in the HEXA cohort. It would have been more interesting if we had been able to predict incident T2D cases using PRS. Also, we did not compare PRS using GWAS summary statistics with and without inclusion of BMI as a covariate.

In summary, this study suggests that PRS can be utilized as a screening strategy for genetically high-risk T2D group. In addition, there is a sub-multiplicative interaction between WC and PRS in T2D and these findings provide the joint etiology of PRS and WC in T2D. Future studies with larger sample size are needed to replicate our findings and examine the characteristics at the extreme end of the PRS distribution in terms of the interaction effect between WC and PRS.

## Methods

### Study population

As a two-stage study, we used two Korean cohorts as a training set to develop genome-wide PRS in the first stage and a test set to evaluate the effectiveness of T2D PRS and perform interaction analysis in the second stage; KARE and HEXA cohorts, respectively. Both cohorts are currently assessed as part of the Korean Genome and Epidemiology Study^24^. We performed the analysis using the data of individuals who had complete information on genetic variations, phenotype, WC, and covariates such as age, sex, and BMI. We used 3294 individuals (745 cases and 2549 controls) from the KARE cohort (age: 40–69 years), which were collected in 2001 from residents in the urban community of Ansan City and the rural community of Anseong City. From the HEXA, which recruited participants aged 40–79 years, 41,387 individuals (5684 cases and 35,703 controls) were used. All studies were approved by the Institutional Review Board of Sookmyung Women's University. The baseline characteristics of the study population in each cohort are summarized in Table 4. The data are publicly available by submission of the application form to Korea Disease Control and Prevention Agency (KDCA) (https://biobank.nih.go.kr).

**Table 4:** Baseline characteristics of the HEXA and KARE cohorts.

	HEXA cohort		
	Cases (n = 5684)	Controls (n = 35,703)	P-value
Age, years	57.901 (7.364)	52.474 (7.929)	0
BMI, kg/m^2^	25.072 (3.081)	23.473 (2.741)	3.67 × 10^–304^
WC, cm	85.384 (8.475)	79.369 (8.392)	0
Sex			1.19 × 10^–166^
Male	2766 (0.487)	10,787 (0.302)	
Female	2918 (0.513)	24,916 (0.698)	

	KARE cohort		
	Cases (n = 745)	Controls (n = 2549)	P-value
Age, years	54.587 (8.558)	49.729 (7.916)	3.67 × 10^–42^
BMI, kg/m^2^	25.678 (3.194)	24.123 (2.870)	2.08 × 10^–33^
WC, cm	86.668 (7.978)	80.750 (8.336)	3.79 × 10^–62^
Sex			0.001
Male	392 (0.526)	1170 (0.459)	
Female	353 (0.474)	1379 (0.541)	0.011
Residence area			
Anseong	393 (0.528)	1208 (0.474)	
Ansan	352 (0.472)	1341 (0.526)	

The mean and standard deviation are shown for continuous variables, and counts and proportions are shown for categorical variables. Ansan and Anseong are the urban and rural communities, respectively. For the KARE cohort, WC was measured three times, and the average value was used. *BMI* body mass index, *WC* waist circumference.

### T2D definition

T2D cases were defined if any one of the following was present: (1) fasting plasma glucose (FPG) ≥ 126 mg/dL, (2) HbA1c level ≥ 6.5%, (3) use of anti-diabetic medications, or (4) history of diagnosed diabetes. In the KARE study, participants had data on 2-h postprandial blood glucose level measurements and the inclusion criteria for T2D cases included 2-h postprandial blood glucose level ≥ 200 mg/dL. Similarly, prediabetes and nondiabetic healthy subjects were defined sequentially and the criteria are presented in Supplementary Table S7. Nondiabetic controls were defined as a subject such that FPG < 100 mg/dL, no medical history of diagnosed T2D, and 2-h postprandial blood glucose level < 140 mg/dL.

### Genotyping

Genomic DNA was extracted from the peripheral blood samples of participants. Genotyping was conducted using Korea Biobank arrays (KoreanChip), which was designed by the Center for Genome Science at the Korea National Institutes of Health. The KoreanChip contains approximately 833,535 SNPs that are specific to the Korean population. The locations of the genes were assigned through the National Center for Biotechnology Information Human Gene Build 37 (hg19). SHAPEIT v2-IMPUTE v2 was used for imputation analysis of genotype data with 1000 Genomes Phase 3 data as a reference panel^24^. Detailed information on the KoreanChip has been reported in a previously published article^25^.

### Polygenic risk scores

PRSs were derived for KARE samples using the imputed genotype data of KARE samples and GWAS summary statistics of Biobank Japan^7^ as weights by PRSice2^15^ software. The PRS of an individual *i* is defined as follows:

$${PRS}_{i}=\sum_{j=1}^{M}{W}_{j}{X}_{ij}$$

where ${X}_{ij}$ is the dosage, expected number of alternative alleles in the* j*-th SNP for an individual *i*, and M is the number of SNPs computed in PRS. ${W}_{j}$ is the weight of the *j*-th SNP, which is the log OR of its association with T2D obtained from GWAS summary statistics of the discovery set. We used Biobank Japan^7^ as the discovery set. PRSs were calculated using P-value thresholds of $\le 5\times {10}^{-8}$, $\le 5.005\times {10}^{-5}$, $\le 1.0005\times {10}^{-4}$ ,… , $\le 0.5$ in steps of $5\times {10}^{-5}$, and the full model including all SNPs ($\le 1$) with LD pruning parameters of ${r}^{2}=0.1$ over 1000-kb windows. The exclusion criteria for SNPs for both Biobank Japan and KARE, which were used for constructing PRS, were as follows: imputation info score < 0.9, minor allele frequency < 0.01 for the discovery and target sets, which correspond to Biobank Japan and KARE, respectively. The explained variance (Nagelkerke pseudo-${R}^{2}$) was derived from a logistic regression model in which PRS was a predictor while controlling for the covariates, compared to a logistic regression model with covariates only. The PRS achieving the maximal explained variance was selected. In our analysis, age, BMI, and sex were considered as covariates and the selected PRS consisted of the 1004 SNPs with P-value threshold of 0.0003 (Supplementary Table S8). To evaluate the PRS constructed from KARE, PRSs were computed using the selected 1004 SNPs for HEXA samples by multiplying the dosage of each SNP by the log of OR from GWAS summary statistics of Biobank Japan^7^. The PRS scores standardized to a mean of 0 and a variance of 1 were used for all analyses.

### Interaction analysis

We investigated multiplicative and additive interactions. Multiplicative interaction was evaluated by performing a likelihood ratio test from the fitting of the logistic regression models both with and without the interaction term. Additive interaction between abdominal obesity and dichotomized PRS was assessed by RERI. Particularly, we dichotomized PRS at the median of the PRS and compared individuals above or equal to the median to those below the median. RERI is expressed using the following formula: RERI = RR_11_ − RR_01_ − RR_10_ + 1, where RR is the relative risk; the reference group consisted of individuals with lower 50% of the T2D genetic risk and without abdominal obesity; RR_01_ represented individuals with lower 50% of T2D genetic risk and with abdominal obesity; RR_10_ represented individuals with upper 50% of T2D genetic risk and without abdominal obesity; and RR_11_ represented individuals with upper 50% of T2D genetic risk and with abdominal obesity.

### Supplementary Information

Supplementary Information 1.

Supplementary Table S8.

## Data Availability

The data analyzed in the study are available by submission of an application form to KDCA (https://biobank.nih.go.kr).
