# Supplementary material for: Risk prediction and interaction analysis using polygenic risk score of type 2 diabetes in a Korean population
Source: Sci Rep. 2024 Mar 21;14:6790. doi: 10.1038/s41598-024-55945-2 (PMC10957984; doi:10.1038/s41598-024-55945-2)
Supplement: Supplementary file 1 — Supplementary Information 1. [file 41598_2024_55945_MOESM1_ESM.docx]

**Risk prediction and interaction analysis using polygenic risk score of type 2 diabetes in a Korean population**

Minsun Song^1*#^, Soo Heon Kwak^2#^, Jihyun Kim^3^

1 Department of Statistics & Research Institute of Natural Sciences, Sookmyung Women's University, Seoul, 04310, Korea

2 Department of Internal Medicine, Seoul National University Hospital, Seoul, 03080, Korea

3 Department of Statistics, Sookmyung Women's University, Seoul, 04310, Korea

*Correspondence and requests for materials should be addressed to M.S. (Email:minsuns@sookmyung.ac.kr)

#These authors contributed equally to this work.


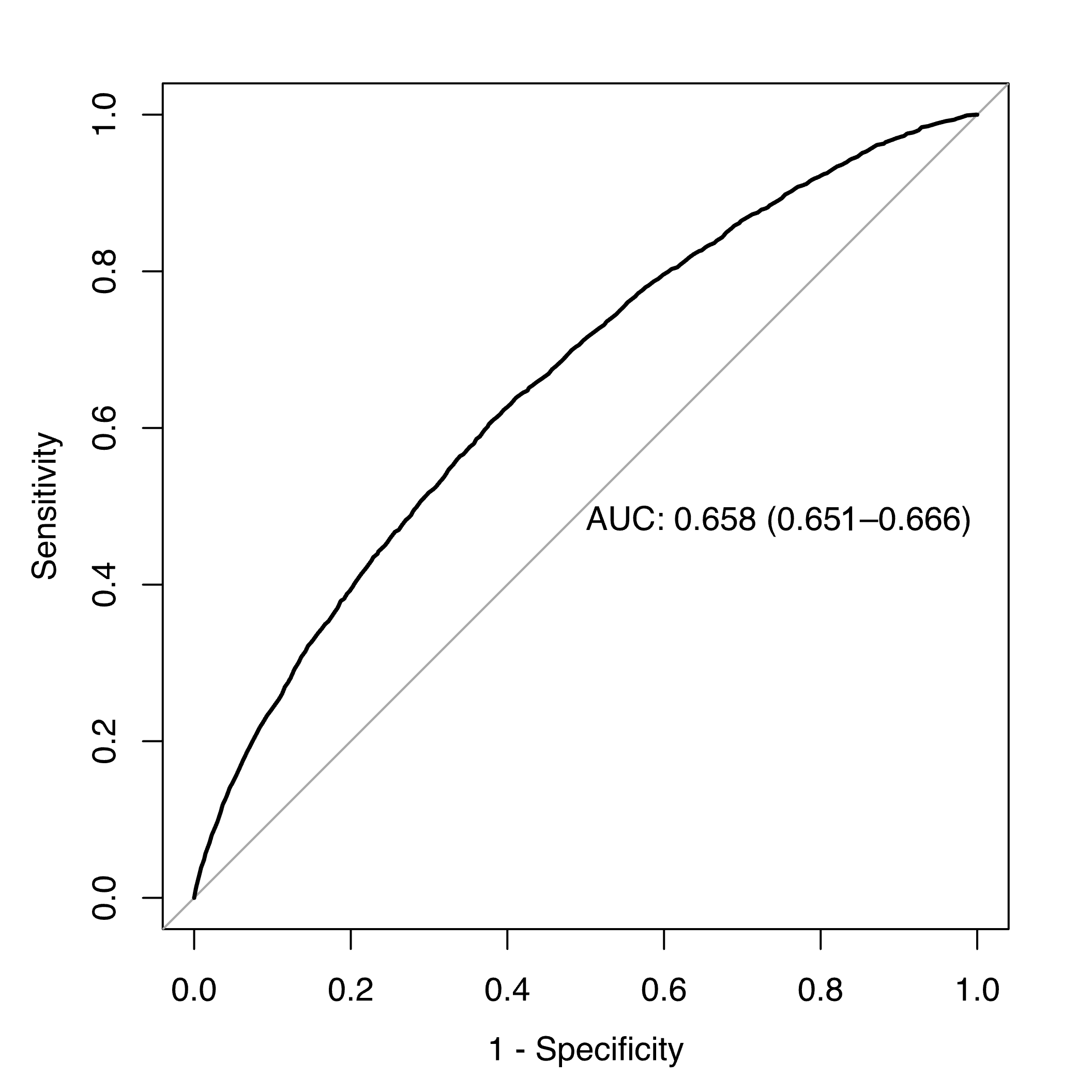

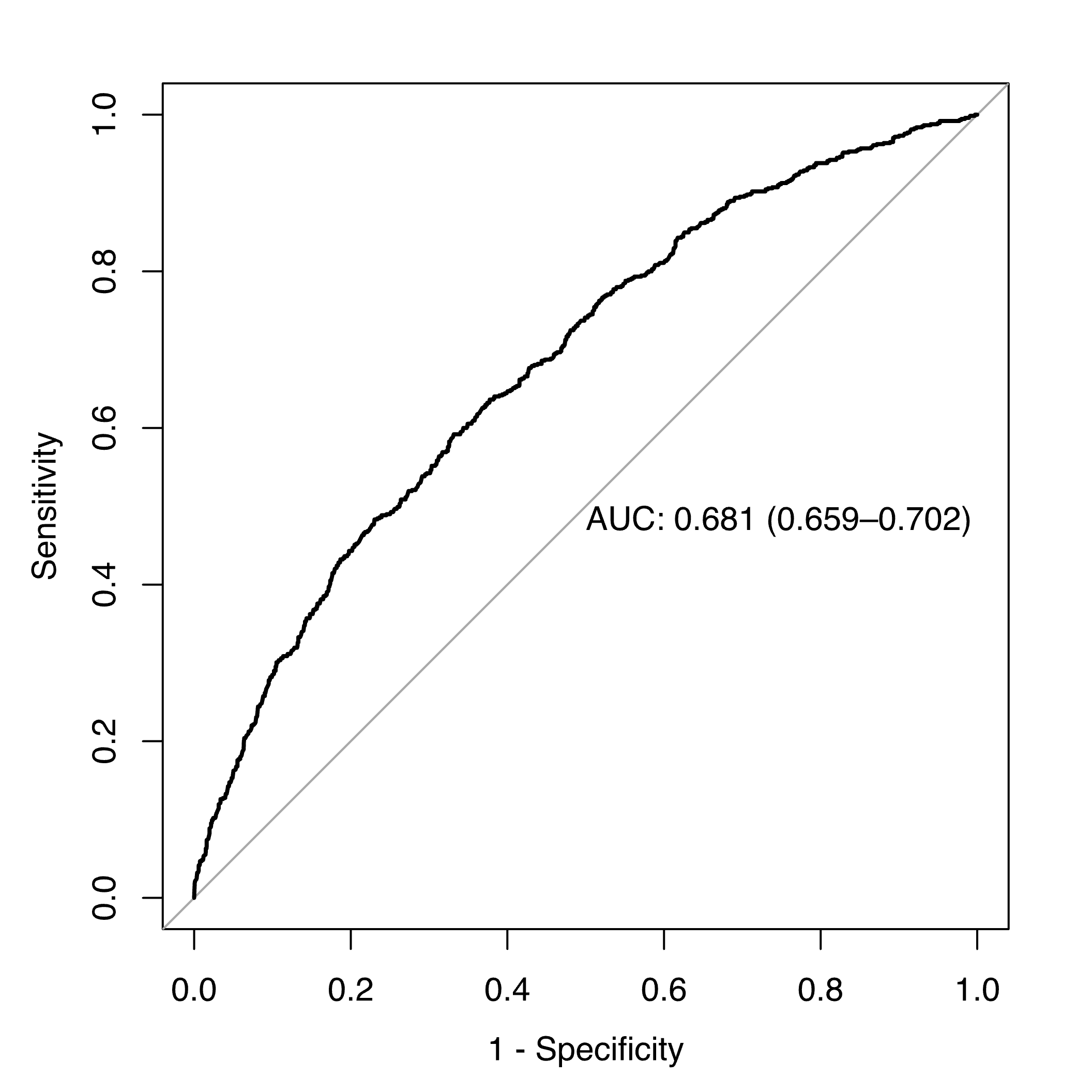


**a**

**b**

Supplementary Figure S1. The AUC of the PRS for the prediction of T2D. (a) The AUC of the PRS in the HEXA cohort was 0.658 (95% CI: 0.650–0.666). (b) The AUC of the PRS in the KARE cohort was 0.681 (95% CI: 0.659–0.702). AUC, area under the receiver operating characteristics curve; PRS, polygenic risk score; CI, confidence interval; T2D, type 2 diabetes.

Supplementary Figure S1. The AUC of the PRS for the prediction of T2D. (a) The AUC of the PRS in the HEXA cohort was 0.658 (95% CI: 0.651–0.666). (b) The AUC of the PRS in the KARE cohort was 0.681 (95% CI: 0.659–0.702). AUC, area under the receiver operating characteristics curve; PRS, polygenic risk score; CI, confidence interval; T2D, type 2 diabetes.


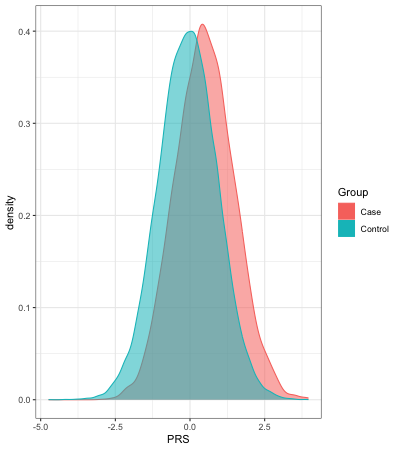

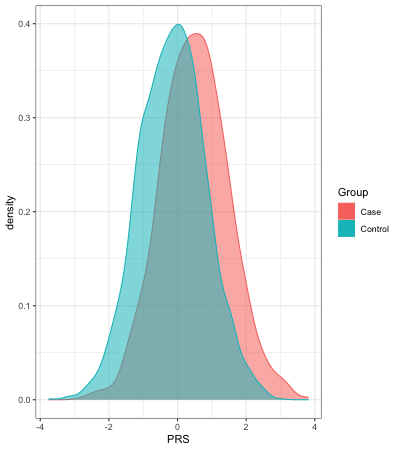


**a**

**b**

Supplementary Figure S2. Comparison of T2D polygenic risk scores between the controls and cases. (a) PRS in HEXA individuals plotted on the x-axis. The means (and SDs) of the PRS among cases and controls were 0.492 (0.981) and -0.078 (0.980), respectively (*p*$\approx$0). (b) PRS in KARE individuals plotted on the x-axis. The means (and SDs) of the PRS among cases and controls were 0.501 (0.982) and -0.146 (0.957), respectively (*p*=2.08$\times$10^-56^). SD, standard deviation; PRS, polygenic risk score; T2D, type 2 diabetes.

Supplementary Table S1. Association between polygenic risk score and risk of T2D in the KARE cohort.

| PRS | Controls | Cases | OR^1^ | 95% CI | P-value |
| --- | --- | --- | --- | --- | --- |
| PRS-ordinal |  |  |  |  |  |
| Q1 (low risk) | 748 | 76 | Ref |  |  |
| Q2 | 665 | 158 | 2.320 | (1.705–3.159) | 8.86$\times$10^-8^ |
| Q3 | 638 | 185 | 3.191 | (2.354–4.327) | $7.70\times$10^-14^ |
| Q4 (high risk) | 498 | 326 | 7.626 | (5.689–10.223) | $4.64\times$10^-42^ |
| P-trend$=1.532\times$10^-48^ | | | | | |
| PRS-continuous | 2549 | 745 | 2.180 | (1.973–2.409) | $5.87\times$10^-53^ |

^1^OR from logistic regression models were adjusted for age, sex, residence area, and BMI. Q, quartile; OR, odds ratio; CI, confidence interval; PRS, polygenic risk score; BMI, body mass index; T2D, type 2 diabetes.

Supplementary Table S2. Risk in high polygenic risk score groups for T2D development in the KARE cohort.

| High PRS group | Reference group | OR^1^ | 95% CI | P-value |
| --- | --- | --- | --- | --- |
| Top 25% | Remaining 75% | 3.648 | (3.013-4.418) | $4.36\times$10^-40^ |
| Top 20% | Remaining 80% | 3.713 | (3.034-4.546) | $4.76\times$10^-37^ |
| Top 15% | Remaining 85% | 4.204 | (3.371-5.243) | $3.42\times$10^-37^ |
| Top 10% | Remaining 90% | 4.308 | (3.330-5.573) | $1.05\times$10^-28^ |
| Top 5% | Remaining 95% | 4.760 | (3.372-6.721) | $7.44\times$10^-19^ |

^1^OR from logistic regression models were adjusted for age, sex, residence area, and BMI. OR, odds ratio; CI, confidence interval; PRS, polygenic risk score; BMI, body mass index; T2D, type 2 diabetes.

Supplementary Table S3. Association between PRS and risk of T2D stratified according to abdominal obesity status in the HEXA and KARE cohorts.

| HEXA | Controls | Cases | OR^1^ | 95% CI | P-value |
| --- | --- | --- | --- | --- | --- |
| With abdominal obesity | 6751 | 2251 | 1.758 | (1.665–1.855) | 0 |
| Without abdominal obesity | 28952 | 3433 | 2.083 | (2.001–2.168) | 0 |
| KARE | Controls | Cases | OR^2^ | 95% CI | P-value |
| With abdominal obesity | 552 | 343 | 1.878 | (1.602–2.201) | $8.22\times$10^-15^ |
| Without abdominal obesity | 1997 | 402 | 2.403 | (2.114–2.731) | 0 |

^1^OR from logistic regression models were adjusted for age, sex, and BMI. ^2^OR from logistic regression models were adjusted for age, sex, residence area, and BMI. OR, odds ratio; CI, confidence interval; PRS, polygenic risk score; T2D, type 2 diabetes; BMI, body mass index.

Supplementary Table S4. Results under the main-effect-only models and under the joint effect model incorporating interaction between PRS and WC in the KARE cohort.

| Model | OR | 95% CI | P-value |
| --- | --- | --- | --- |
| Main-effect-only model |  |  |  |
| WC | 1.070 | (1.050–1.090) | 7.06$\times$10^-13^ |
| PRS | 2.180 | (1.973–2.409) | 5.87$\times$10^-53^ |
| Joint effect model incorporating interaction |  |  |  |
| Interaction between WC and PRS | 0.987 | (0.975–0.999) | 0.040 |

The main-effect-only model for WC includes age, sex, BMI, residence area, and WC. The main-effect-only model for PRS includes age, sex, BMI, residence area, and PRS. The joint effect model incorporating interaction includes age, sex, BMI, residence area, WC, PRS, and the interaction term between PRS and WC. The WC was measured three times, and the average value was used. OR, odds ratio; CI, confidence interval; PRS, polygenic risk score; WC, waist circumference; BMI, body mass index.

Supplementary Table S5. AUC for the PRS in the HEXA and KARE cohorts.

| Cohort | All | | With abdominal obesity | | | Without abdominal obesity | | |  |  |
| --- | --- | --- | --- | --- | --- | --- | --- | --- | --- | --- |
|  | AUC | 95% CI | | AUC | 95% CI | AUC | | 95% CI | | |
| HEXA |  |  | |  |  |  | |  | | |
| All | 0.658 | (0.651-0.666) | | 0.630 | (0.616-0.643) | 0.679 | | (0.670-0.688) | | |
| Men | 0.657 | (0.646-0.668) | | 0.625 | (0.606-0.644) | 0.681 | | (0.667-0.695) | | |
| Women | 0.664 | (0.654-0.675) | | 0.639 | (0.621-0.657) | 0.682 | | (0.669-0.694) | | |
| KARE |  |  | |  |  |  | |  | | |
| All | 0.681 | (0.659-0.702) | | 0.637 | (0.600-0.673) | 0.714 | | (0.686-0.741) | | |
| Men | 0.692 | (0.662-0.722) | | 0.631 | (0.569-0.694) | 0.717 | | (0.683-0.752) | | |
| Women | 0.673 | (0.641-0.704) | | 0.643 | (0.597-0.688) | 0.720 | (0.673-0.766) | | |  |

AUC, Area under the receiver operating characteristic curve; CI, confidence interval; PRS, polygenic risk score.

Supplementary Table S6: AUC estimates and 95% confidence intervals for the HEXA cohort with the various prediction models.

| Model | AUC (95% CI) |
| --- | --- |
| PRS | 0.658 (0.651 - 0.666) |
| WC | 0.694 (0.687 - 0.701) |
| WC+PRS | 0.750 (0.743 - 0.756) |
| WC+AGE+SEX+BMI | 0.747 (0.741 - 0.754) |
| WC+AGE+SEX+BMI+PRS | 0.794 (0.788 - 0.800) |

AUC, Area under the receiver operating characteristic curve; CI, confidence interval; PRS, polygenic risk score.

Supplementary Table S7. Criteria for the definition of T2D case, prediabetic subject, and non-diabetic control.

|  | T2D case | Prediabetic subject | Control |
| --- | --- | --- | --- |
| FPG | ≥126 mg/dL | 100mg/dL–125 mg/dL | <100 mg/dL |
| HbA1c | ≥6.5 % | 5.7%–6.4% | <5.7% |
| Using anti-diabetic medications | Yes | No | No |
| History of diagnosed diabetes | Yes | No | No |
| 2-h glucose | ≥200mg/dL | 140mg/dL–199 mg/dL | <140 mg/dL |

T2D case, prediabetic subject and non-diabetic healthy subject were defined sequentially. T2D case was defined as FPG≥126 mg/dL, HbA1c level≥6.5%, the use of anti-diabetic medications, history of diagnosed diabetes, or 2-h postprandial blood glucose level ≥ 200 mg/dL. Prediabetes was defined as 100 mg/dL$\leq$FPG$\leq$125mg/dL, 5.7% $\leq$HbA1c level$\leq$6.4%, or 140 mg/dL$\leq$2-h postprandial blood glucose level$\leq$199 mg/dL among subjects who were not defined as T2D case. Control was defined as FPG <100 mg/dL, 2-h postprandial blood glucose level <140 mg/dL, and no medical history of diagnosed T2D among subjects who were not defined as T2D case or prediabetes. FPG, fasting plasma glucose; HbA1c, hemoglobin A1c; T2D, type 2 diabetes.
